# Processing Optimization for Halbach Array Magnetic Field-Assisted Magnetic Abrasive Particles Polishing of Titanium Alloy

**DOI:** 10.3390/ma17133213

**Published:** 2024-07-01

**Authors:** Jia Qin, Ming Feng, Qipeng Cao

**Affiliations:** 1 Faculty of Optoelectronic Manufacturing, Zhejiang Industry & Trade Vocational College, Wenzhou 325035, China; qinjia@zjitc.edu.cn; 2 College of Mechanical and Electrical Engineering, Wenzhou University, Wenzhou 325035, China; fming@wzu.edu.cn; 3 Ruian’s Graduate School, Wenzhou 325035, China; 4 Wenzhou Polytechnic, Wenzhou 325035, China

**Keywords:** Halbach array, magnetic abrasive particles, titanium alloy, response surface model

## Abstract

To extend the working life of products made of titanium alloy, it is necessary to improve the polishing method to diminish the remaining defects on the workpiece surface. The Halbach array-assisted magnetic abrasive particle polishing method for titanium alloy was employed in this work. The distribution of magnetic field strength was simulated and verified at first to learn the characteristics of the Halbach array used in this work. Then, the polishing performance of the polishing tool was studied by conducting the polishing test, which aimed to display the relationship between shear force and surface roughness with polishing time, and the surface morphology during polishing was also analyzed. Following the establishment of the response surface model, a study on the optimal polishing parameters was conducted to obtain the suitable parameters for maximum shear force and minimum surface roughness. The results show that the maximum shear force 6.11 N and minimum surface roughness *S*_a_ 88 nm can be attained, respectively, under the conditions of (1) polishing tool speed of 724.254 r·min^−1^, working gap of 0.5 mm, and abrasive particle size of 200 μm; and (2) polishing tool speed of 897.87 r·min^−1^, working gap of 0.52 mm, and abrasive particle size of 160 μm.

## 1. Introduction

Titanium alloys are widely used in superconducting, aerospace, and biomedical fields due to their excellent comprehensive properties. In low-temperature environments, titanium alloys exhibit superior performance, such as good low-temperature toughness, high specific strength, and non-magnetic properties [1,2,3]. In high-temperature environments, titanium alloys still maintain good high low-cycle fatigue strength and corrosion resistance due to their low thermal expansion, low thermal conductivity, and low modulus characteristics [4,5,6]. Compared to high-temperature materials such as alloy steel, stainless steel, and high-temperature alloys, titanium alloys meet stringent requirements for strength, corrosion resistance, and fatigue resistance while having a lower density, which reduces the impact of material weight on equipment when used as structural components [7,8]. Thus, titanium alloys are extensively utilized in the manufacture of aircraft engine blades, bio-implantable devices, and other applications.

The traditional method for polishing titanium alloys relies on the micro-grinding effect of abrasives under mechanical and frictional forces. Traditional mechanical polishing is mainly used for the finishing of titanium alloy disks and blades in aerospace engines, which can eliminate milling marks on the machined surface. However, due to the low thermal conductivity of titanium alloys, it is difficult for coolant to reduce the high temperature in the internal polishing area. Therefore, when the instantaneous temperature in local areas is high, the titanium alloy surface is burned and the subsurface microstructure can also be altered, particularly causing residual tensile stress on the polished surface and subsurface of the titanium alloy, which can also affect its service life. To address the issues associated with traditional polishing processes, various new surface polishing techniques have emerged. Among them, chemical polishing often uses toxic substances such as hydrofluoric acid and chromic acid to prepare the polishing solution, resulting in residual substances adhering to the alloy surface after polishing. Electrolytic polishing involves immersing the workpiece in an electrolyte solution and removing surface defects through electrolysis reactions. However, the electrolyte used in single-electrolyte polishing is usually strongly acidic or alkaline, and improper operation can easily endanger the health of experimenters. Laser polishing technology is an emerging surface processing technique that achieves surface polishing by irradiating the workpiece with a laser, but it requires expensive equipment and a high-precision control system for technical support. As a flexible polishing method, magnetic field-assisted polishing possesses advantages such as high conformability of the flexible polishing head with any curved surface, small subsurface damage layers, and low polishing temperatures [9,10,11,12], making it highly suitable for ultra-precision polishing of titanium alloy workpieces. Wang et al. [13] stacked two identical ring-shaped permanent magnets in the order of N-S-N-S and fixed the magnet rotor with a certain eccentricity from the fluid plate, enabling the magnetic field strength to remain constant during magnet rotation while the magnetic field lines undergo periodic motion to generate a spatially dynamic magnetic field. Through polishing experiments, the optimal parameters of the dual-magnetic-field polishing device were determined, enhancing the polishing ability of the magnetic compound fluid (MCF) polishing tool under the dual magnetic field. Wu et al. [14] generated a rotating magnetic field by eccentrically rotating the magnet, prompting periodic refreshment of the carbonyl iron particles (CIP) and abrasive particles (AP) in the polishing area, thereby extending the working life of the polishing tool and improving polishing efficiency to a certain extent. Although the magneticrheological polishing method can achieve high-precision and high-quality polishing results, compared to traditional polishing methods, it lacks an external pressure device to provide a significant polishing force. The force exerted by the abrasive particles on the workpiece solely relies on the magnetic chain structure and magnetic field intensity, and the abrasive particles are in a free state with weak control capability of the magnetic chain. Therefore, traditional magneticrheological polishing has a low polishing efficiency. To improve polishing efficiency, scholars have proposed a new technique that bonds magnetically sensitive particles and abrasive particles together using a binder for polishing. Magnetic abrasive technology achieves polishing of the workpiece through the cooperation of an external magnetic field and magnetic abrasive particles, attracting together magnetic abrasive particles with abrasive properties. The magnetic abrasive particles are changed into magnetic brushes that perform cutting, scratching, and other motions relative to the workpiece surface for removing materials. Zhu et al. [15] investigated the influence of the composite magnetic circuit on the surface quality and surface roughness in magnetic abrasive polishing, addressing the issue of low processing efficiency caused by the small magnetic force acting on the abrasive particles. The improvement rate of surface roughness increased by 40%. Focusing on the challenge of precision machining of TC4 titanium alloy surfaces [16,17,18], Zhao Yang et al. [19] added cylindrical auxiliary magnetic pole polishing under the action of an electromagnetic field to improve the internal surface quality of TC4 titanium alloy tubes with the magnetic abrasive polishing method.

**Figure 1:**
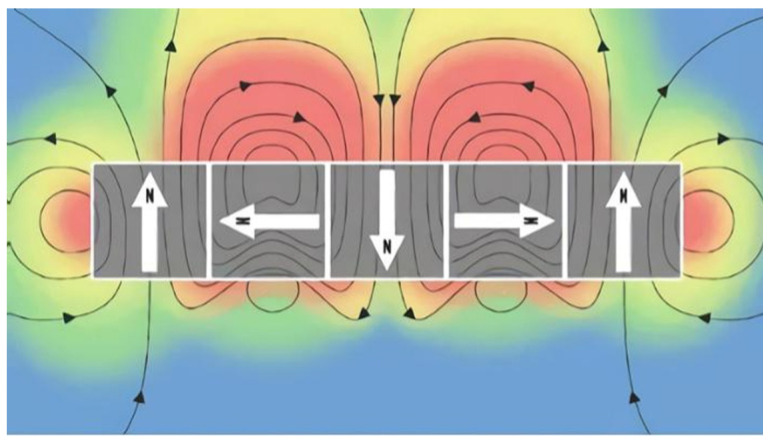
Schematic diagram of the Halbach array.

The key to magnetic abrasive polishing technology lies in the excellent magnetic field intensity and magnetic field distribution, and the type of magnetic source structure is an important factor that guarantees the polishing force of magnetic abrasive particles. Zhou Qinqin et al. [20] designed three magnetic field generation devices based on electromagnets and studied the magnetic field intensity and effective polishing area of the magnetic field generation devices through simulation and experimental analysis. It was found that the ring-shaped magnetic field generation device had high polishing efficiency and the best polishing results. Singh [21] achieved magnetic abrasive particle polishing of 3D surfaces using electromagnetic fields. Although the magnetic field intensity of the electromagnetic field can be controlled by adjusting the current, the electromagnetic field has unsolvable issues such as large device volumes and severe heat generation during operation. Meanwhile, with the rapid development of permanent magnetic materials and the emergence of rare-earth strong magnetic materials, more and more scholars have turned to the design and research of permanent magnetic fields. Verma [22] designed a mutually exclusive permanent magnetic field to enhance the magnetic field intensity and conducted experimental research on the inner wall of stainless steel (SS304) tubing, ultimately obtaining a surface roughness of 56 nm. The Halbach array is formed by arranging permanent magnet units with different magnetization directions according to a certain pattern, with each of the four permanent magnet units constituting a period. Figure 1 shows the magnetic flux density distribution contour of the Halbach permanent magnet array, with the arrow indicating the magnetization direction of the permanent magnet unit. If the end effect is ignored, the generated magnetic field after the array has a weak magnetic field on one side and a strong magnetic field on the other side, and the strong side has a higher magnetic flux density and better air gap magnetic field distribution. Guo et al. [23] proposed a planar magnetorheological polishing method using a linear Halbach array, which improved the efficiency of planar magnetorheological polishing. The material removal rate for K9 optical glass can reach 1.38 mm^3^/min, and an ultra-smooth surface with a surface roughness of less than *R*_a_ 1 nm can be obtained after 30 min of polishing. Liu Le et al. [24] designed a magnetic source structure based on the Halbach array, improving the polishing efficiency of magnetic abrasive particles on 30CrMnSi high-strength structural steel. In summary, the Halbach array magnetic field can solve the problems of weak magnetic field intensity and low magnetic energy utilization in traditional permanent magnetic field source structures. Currently, Halbach array magnetic field-assisted magnetic abrasive particle polishing is mainly applied to precision polishing of optical glass, high-strength steel, and ceramic materials, but research on titanium alloy polishing has not yet been conducted in depth.

To conduct research on precision polishing of titanium alloy materials using Halbach array magnetic field-assisted magnetic abrasive particles, this paper proposes a Halbach array-assisted magnetic abrasive polishing method that combines the high polishing efficiency of magnetic abrasive particles with the stronger magnetic field of the Halbach array in the polishing area. A circular Halbach array magnetic field is designed, and the order of influence and interaction effects of polishing tool speed, abrasive particle size, and working gap on the titanium alloy polishing shear force and surface roughness are studied through response surface methodology and variance analysis. A linear regression prediction model is established, and an optimal combination of polishing parameters within a certain range is obtained.

## 2. Materials and Methods

### 2.1. Principle and Device

**Figure 2:**
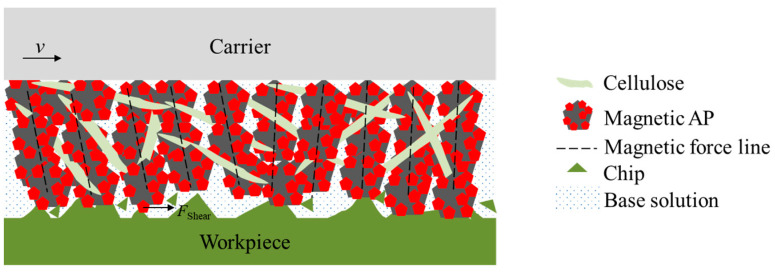
Schematic diagram of the magnetic abrasive particle polishing method.

**Figure 3:**
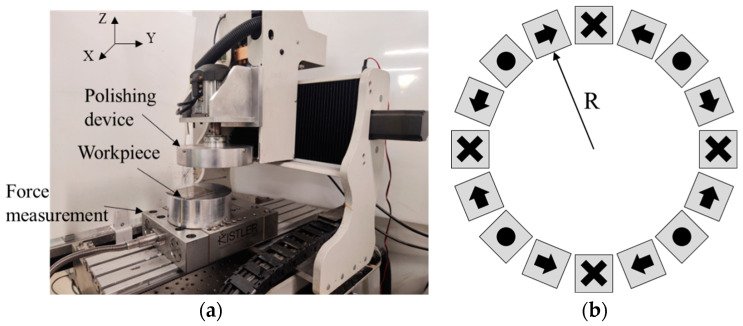
Experimental device and Halbach array magnetic field employed in this work. (**a**) polishing device; (**b**) arrangement of Halbach array.

The principle of the magnetic abrasive particle polishing method used in this paper is illustrated in Figure 2. An appropriate volume of a mixture containing magnetic abrasive particles, cellulose, and deionized water is added outside the carrier. Under the action of the magnetic field, magnetic chains are formed along the magnetic field lines. The cellulose connects the magnetic chains together, enhancing the strength and toughness of the magnetic chains and keeping them stronger from being destroyed. The workpiece material is subjected to the shear force of the magnetic abrasive particles, removing the surface roughness peaks of the workpiece and gradually improving the surface quality. When the gap between the carrier and the workpiece is set to the preset working gap *h*, the carrier rotates at a speed of *n*_c_ to polish the workpiece surface. In this paper, a Halbach array is composed of 16 N38 neodymium-boron permanent magnets. The experimental setup, which was provided by Shenzhen ultra-nano precision Technology Co., Ltd. (Shenzhen, China, repeated positioning accuracy 0.5 μm, spindle speed 0–6000 rpm with 0.1 rpm resolution), is shown in Figure 3a, where the polishing device is installed on the spindle of a three-axis motion platform and the workpiece is mounted on the XY platform. The arrangement of the Halbach array, including the direction of magnetization and diameter (*R*) of the magnet array, which also go through the center line of a magnet, is depicted in Figure 3b.

### 2.2. Experimental

To enhance the accuracy and reliability of the process parameter levels and interactions during the experimental process, it is necessary to conduct pretreatment and post-treatment on the titanium alloy test specimens. Firstly, a 10,000-grit sandpaper is used to grind the test specimens to remove the surface oxide layer. Then, the test specimens are placed in a sealed bag, immersed in anhydrous ethanol, and sealed for 30 min of ultrasonic cleaning to remove surface impurities. Finally, an OLYMPUS OLS4100 laser confocal microscope is used to observe the surface morphology of the processed area of the test specimens and measure the surface roughness. During the experiment, a Kistler 9139A (Fy −4.2 pC/N, Fx, Fz −8.2 pC/N, Kistler Co., Ltd., Bern, Switzerland) is used to measure the shear force. After the experiment, the workpiece is immersed in deionized water and undergoes ultrasonic cleaning to remove residual impurities on the surface after the polishing. Subsequently, the surface morphology of the processed area is observed, and the surface roughness is measured.

A TC4 titanium alloy with a specified size of 100 mm × 100 mm × 3 mm is used as the polishing specimen, and the process parameters mainly include the polishing tool speed, working gap, and magnetic abrasive particle size. Based on the exploration of preliminary process parameters, it has been determined that the polishing tool speed (*n*_c_), working gap (*h*), and abrasive particle size (*r*) have a significant impact on the experimental results. Through single-factor experiments, it is determined that the polishing tool speed (*n*_c_) of 600 r·min^−1^, working gap (*h*) of 1 mm, and abrasive particle size (*r*) of 150 μm achieve good polishing effects and efficiency. Due to the discontinuous values of the test factors, the Box-Behnken Design (BBD) of the response surface methodology is adopted to design the experiment. The response surface experiment is designed with the polishing tool speed (*A*), working gap (*B*), and abrasive particle size (*C*) as independent variables. The response surface test factors and their levels are shown in Table 1. The composition of the magnetic abrasive particle slurry used in this work is shown in Table 2. The magnetic abrasive particles and cellulose (~150 μm) were supplied by Jiangsu Tianyi Ultra-fine Metal Powder Co., Ltd., Jiangsu, China.

## 3. Results and Discussion

### 3.1. Magnetic Field Simulation

**Figure 4:**
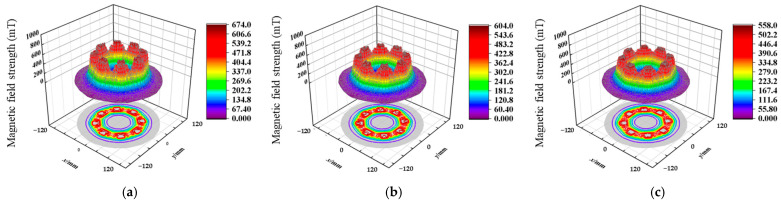
Simulation of magnetic field strength at different working gaps *h*: (**a**) *h* = 0.5 mm, (**b**) *h* = 1 mm, and (**c**) *h* = 1.5 mm.

The magnetic field intensity directly determines the magnetic force exerted on the magnetic abrasive particles. The magnetic field of the Halbach array was simulated using COMSOL software, and the validity of the simulation was verified through measurement comparisons. Simulation calculations of the magnetic flux density module were performed on the planar areas with working gaps of *h* = 0.5 mm, *h* = 1 mm, and *h* = 1.5 mm. The simulation results are shown in Figure 4. The HM100 Tesla meter (resolution 0.1 mT, Huaming Instrument Equipment Co., Ltd., Zhengzhou, China) was used to measure the tangential and radial components of the magnetic field. The magnitude of the magnetic flux density, |*B*|, at any spatial location can be calculated based on its tangential and radial components by using the following formula:

(1) $$\left|B\right|=\sqrt{{B_{r}}^{2}+{B_{t}}^{2}}$$

where |*B*| represents the magnitude of the magnetic flux density, *B_t_* is the tangential component of the magnetic flux density, and *B_r_* is the radial component of the magnetic flux density.

**Figure 5:**
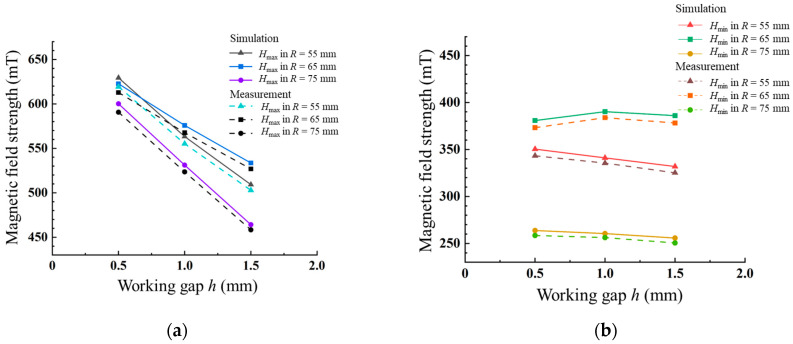
(**a**) Maximum and (**b**) minimum magnetic field strength of the Halbach array simulated and measured at different working gaps *h*.

As shown in the simulation results in Figure 4, the magnetic field generated by the Halbach array exhibits periodic changes in magnetic field strength on the plane of the reset working gap. The Halbach array can produce a stronger magnetic field on a plane at a smaller working gap, with a maximum magnetic field strength of 674 mT when *h* = 0.5 mm. As the working gap increases, the magnetic field strength generated by the Halbach array gradually decreases, and the maximum magnetic field strength drops to 558 mT when *h* = 1.5 mm. Figure 5 presents the simulation and measured values of the maximum and minimum magnetic field strength on the circumference with different array diameters *R*. *R* = 55 mm represents the inner diameter of the magnetic field array; *R* = 65 mm represents the middle diameter of the magnetic field array, which is the circumference diameter where the magnet centers are located; and *R* = 75 mm represents the outer diameter of the magnetic field array. As the radial diameter *R* increases, the maximum magnetic field strength gradually decreases at the same working gap, and the rate of decrease continuously increases. Moreover, as the working gap continuously increases, the rate of decrease in the maximum magnetic field strength also accelerates. As the radial diameter *R* increases, the minimum magnetic field strength gradually decreases at the same working gap, and the rate of decrease continuously increases. As the working gap continuously increases, the minimum magnetic field strength first increases and then decreases at the circumference *R* = 65 mm, where the magnet centers are located. Under other working gaps, the minimum magnetic field strength shows a decreasing trend. However, the minimum magnetic field strength does not change significantly compared to the maximum magnetic field strength under different working gaps.

### 3.2. Polishing Performance

**Figure 6:**
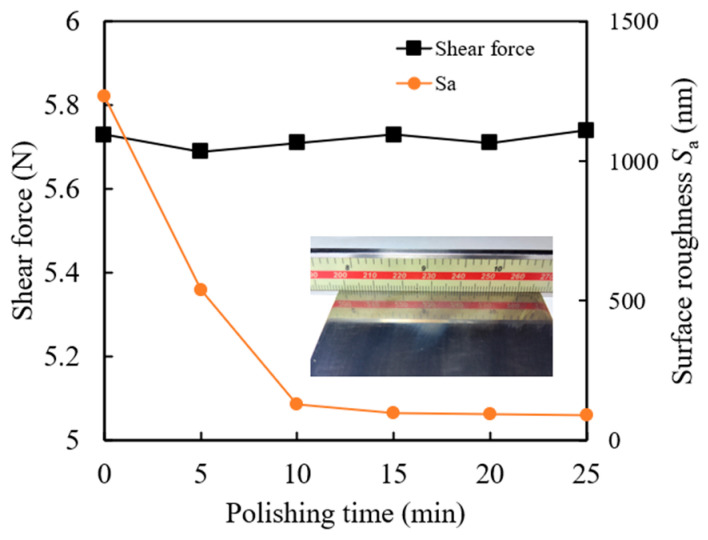
Effect of polishing time on shear force and surface roughness.

**Figure 7:**
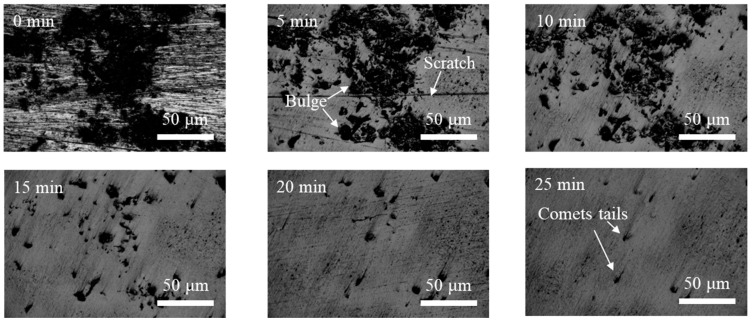
Surface morphology at different polishing times.

Under experimental conditions of *n*_c_ = 900 r·min^−1^, working gap *h* = 0.5 mm, and abrasive particle size = 150 μm, a polishing test was conducted on the titanium alloy workpiece with an initial surface roughness *S*_a_ of 1.2 μm for 25 min. The shear force and surface roughness were measured every 5 min. Figure 6 shows the variation of shear force and surface roughness with different polishing times. It can be observed that the shear force does not change significantly with increasing polishing time, fluctuating around 5.72 N. Therefore, the polishing time has little effect on the shear force during the polishing, indicating a stable material removal capability of the magnetic polishing tool. Additionally, the surface roughness decreases continuously as the polishing time increases. During the first 10 min, the surface roughness *S*_a_ rapidly decreases, while it gradually stabilizes in the subsequent 15 min. After 20 min of polishing, the surface roughness *S*_a_ reaches its optimal value. Figure 7 shows a typical evolution of the workpiece surface morphology at different polishing times. During the first 10 min, significant changes occur on the surface as the initial grinding marks are quickly removed, but the obvious scratch and bulge still remain after 5 min polishing. As the polishing time increases, the rougher areas gradually become smoother, and scratches are diminished. By 25 min, most protrusions are removed. However, comet-tail polishing marks remain on the workpiece surface. These marks are due to the uneven dispersion and agglomeration of some magnetic abrasive particles, which form coarse magnetic chains under the influence of the magnetic field. These magnetic chains can generate large micro-cutting indentation depths and leave comet-tail-shaped surface defects on the workpiece surface during relative motion [25].

### 3.3. Response Surface Method Analysis

The Box-Behnken Design (BBD) experimental groups and test results are shown in Table 3 for analysis in the following work. Taking the shear force *F* (*x*) and surface roughness (*y*) as the response values, a multivariate regression equation model is established between the different response values (*x*, *y*) and factors (*A*, *B*, *C*). The binary interactions between the factors, such as the interaction between polishing tool speed and working gap (*A* × *B*), polishing tool speed and abrasive particle size (*A* × *C*), and working gap and abrasive particle size (*B* × *C*), are considered and analyzed. The following mathematical prediction models have been established:

(2) $$\begin{array}{l} F=5.5025-0.001963\times A-2.4555\times B+0.009555\times C\\ \;\;\;\;+0.000033\times A\times B-3.33333\times 10^{-7}\times A\times C+0.0107\times B\times C\\ \;\;\;\;+2.30278\times 10^{-6}\times A^{2}+1.599\times B^{2}+0.000022\times C^{2} \end{array}$$

(3) $$\begin{array}{l} Sa=44.5+0.324833\times A+213.15\times B-0.9615\times C\\ \;\;\;\;\;-0.003333\times A\times B-0.000233\times A\times C+0.63\times B\times C\\ \;\;\;\;\;-0.000269\times A^{2}-123.7\times B^{2}+0.00103\times C^{2} \end{array}$$

(4) $$Re.=a_{0}+a_{1}\times A+a_{2}\times B+a_{3}\times C+a_{4}\times A\times B+a_{5}\times A\times C+a_{6}\times B\times C+a_{7}\times A^{2}+a_{8}\times B^{2}+a_{9}\times C^{2}$$

Equation (4) [26] was used for calculating the mathematical prediction models, where *R_e_*. was the target result and a_0_–a_9_ were coefficients, respectively.

#### 3.3.1. Variance Analysis of the Fitting Model for Shear Force

The variance analysis results of the fitting equation for the shear force *F* are presented in Table 4. The *p*-value of the mathematical prediction model for the fitting equation indicates the significance of the model, whereas a smaller *p*-value suggests a more significant model [27]. Typically, a *p*-value of ≤0.05 is required for the model to be considered significant at a 95% confidence level [28]. Additionally, to ensure the accuracy of the mathematical prediction model, it is also necessary that the *p*-value for the lack of fit term be >0.05. Based on the variance analysis results in Table 4, the *p*-values for the *AB* and *AC* terms in the shear force fitting equation are much greater than 0.1, indicating that they have an insignificant effect on the equation. This is due to the equal proportional relationship between the values of polishing tool speed (*A*) and working gap (*B*), specifically 300:0.5 = 600:1 = 900:1.5, which affects the accuracy and significance of the equation fitting and causes some related experimental data. Additionally, the *p*-value for the *C*^2^ term is greater than 0.05, indicating that the *C*^2^ term has a slightly insignificant effect on the shear force fitting equation. This is because as the abrasive particle size increases, the shear force *F* (monotonically) increases, resulting in the insignificance of the *C*^2^ term in the equation. To obtain a more accurate and reliable fitting equation, the *AB*, *AC*, and *C*^2^ terms were removed from the original equation, and the experimental data were re-fitted to obtain an optimized shear force *F* fitting equation:

(5) $$F=5.06586-0.002018\times A-2.45855\times B+0.015925\times C\;-0.0107\times B\times C+2.3348\times 10^{-6}\times A^{2}+1.61053\times B^{2}$$

The results of the variance analysis for the optimized shear force fitting equation are shown in Table 5. The variance analysis results in Table 5 indicate that the *p*-value of the shear force model is <0.0001, and the *p*-values of all terms in the model are <0.05. The *p*-value of the lack-of-fit term is 0.4455 > 0.05, indicating that the mathematical prediction model constructed based on the experimental results has excellent significance. To verify the reliability, accuracy, and effectiveness of the optimized fitting equation of the regression model, essential indicators such as the correlation coefficient *R*^2^, adjusted correlation coefficient *R*^2^*_Adj_*, predictive correlation coefficient *R*^2^*_Pre_*, coefficient of variation *C.V.*, and signal-to-noise ratio (*SNR*) were analyzed. The closer the correlation coefficient *R*^2^ is to 1, the better the fitting results of the model fit. A difference of <0.2 between the adjusted correlation coefficient *R*^2^*_Adj_* and the predictive correlation coefficient *R*^2^*_Pre_* indicates a better degree of model conformity. A variation coefficient of *C.V.*% < 10% suggests good model stability. An SNR > 4 indicates sufficient signal and high credibility of the model data [29].

As to Table 6, the optimized correlation coefficient *R*^2^ is 0.9392, indicating that the model can explain 93.92% of the data. The gap between the correlation coefficient *R*^2^ and the adjusted correlation coefficient *R*^2^*_Adj_*, as well as the gap between *R*^2^*_Adj_* and the predictive correlation coefficient *R*^2^*_Pre_*, has narrowed. Moreover, *R*^2^*_Adj_* is closer to 1, suggesting an improvement in model prediction after optimization. The variation coefficient has slightly decreased, indicating a marginal enhancement in model stability. Additionally, the small increase in the signal-to-noise ratio (*SNR*) suggests an improvement in the credibility of the model’s data. In summary, the model demonstrates good agreement with the actual situation, and it can be used to analyze the experimental data.

In the optimized shear force fitting model, the *p*-value indicates whether a factor has a significant effect on the response target, while the *F*-value represents the influence degree of the factor on the response target; the larger the *F*-value, the more significant the factor’s impact on the response target [30]. Among the linear terms, the *p*-value of the working gap (*B*) is <0.0001, indicating it is an extremely significant factor. The *p*-values of the polishing tool speed *n*_c_ (*A*) and abrasive particle size *r* (*C*) are both less than 0.05, indicating they are significant factors. Because the *F*-values are 18.91, 60.76, and 23.37, respectively, the significance order of the impact of processing parameters on the shear force is: working gap (*B*) > abrasive particle size *r* (*C*) > polishing tool speed *n*_c_ (*A*).

#### 3.3.2. Variance Analysis of the Fitting Model for Surface Roughness

The results of the variance analysis for the surface roughness *S_a_* fitting equation are shown in Table 7. According to the variance analysis results, the *p*-values of the *AB* term, *AC* term, and *C*^2^ term in the surface roughness fitting equation are all greater than 0.1, indicating that these terms are not significant for the surface roughness fitting equation. This is because as the working gap increases, the surface roughness *S_a_* increases monotonically, resulting in the insignificance of these terms in the surface roughness fitting equation. To obtain a more accurate and reliable fitting equation, insignificant terms such as the *AB* term, *AC* term, and *C*^2^ term are removed from the fitting equation. The experimental data is then fitted again to obtain an optimized surface roughness (*S_a_*) fitting equation:

(6) $$\begin{array}{c} Sa=46.49342+0.284693\times A+210.06579\times B-0.7925\times C\\ +0.63\times B\times C-0.000267\times A^{2}-123.15789\times B^{2} \end{array}$$

The variance analysis for the optimized surface roughness fitting equation is shown in Table 8. According to the variance analysis in Table 8, the *p*-value of the surface roughness model is <0.0001, and the *p*-values of all terms in the model are <0.05, indicating that the mathematical prediction model constructed based on the experimental results has good significance. The credibility analysis of the surface roughness prediction model is presented in Table 9. The optimized correlation coefficient *R*^2^ = 0.9565, indicates that the model can explain 95.65% of the data. The gap between the correlation coefficient *R*^2^ and the adjusted correlation coefficient *R*^2^*_Adj_*, as well as the gap between *R*^2^*_Adj_* and the predicted correlation coefficient *R*^2^*_Pre_*, has narrowed, and *R*^2^*_Pre_* is closer to 1, suggesting that the fit degree of the optimized model has been improved. The coefficient of variation has slightly decreased, indicating a slight increase in model stability. The signal-to-noise ratio has increased slightly, indicating an improvement in the credibility of the model data. Therefore, the model fits the actual situation well and can be used to analyze the experiments. In the optimized surface roughness fitting model, the *p*-value of the working gap *h* (*B*) is <0.0001, indicating that it is a highly significant factor; the *p*-values of the polishing tool speed *n*_c_ (*A*) and abrasive particle size *r* (*C*) are both less than 0.05, indicating that they are significant factors. The *F*-values are 12.67, 92.98, and 7.24, respectively, which indicates that the significance order of the impact of processing parameters on surface roughness is: working gap *h* (*B*) > polishing tool speed *n*_c_ (*A*) > abrasive particle size *r* (*C*).

### 3.4. Interaction of Processing Parameters

#### 3.4.1. Influence on Shear Force

**Figure 8:**
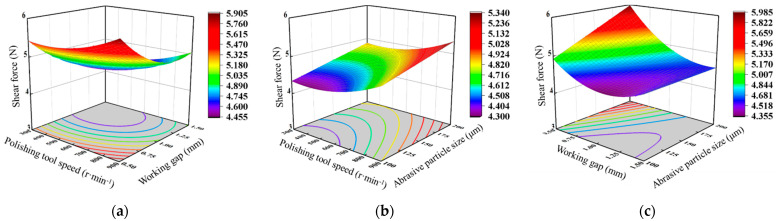
Response surface of the effect of processing parameters on shear force: (**a**) polishing tool speed and working gap; (**b**) polishing tool speed and abrasive particle size; (**c**) working gap and abrasive particle size.

Figure 8 represents the interaction of the polishing tool speed, working gap, and abrasive particle size on the shear force. Figure 8a shows the response surface of the interaction between the polishing tool speed and the working gap on the shear force when the abrasive particle size is 150 μm. It can be observed that under different polishing tool speeds, as the working gap increases, the shear force rapidly decreases and then slightly increases, and the larger the polishing tool speed, the greater the amplitude of the change in shear force with the working gap. This indicates that the greater the polishing tool speed, the greater the influence of the working gap on the shear force. This is because as the polishing tool speed increases, the centrifugal force acting on the magnetic chains formed by the magnetic particles in the fluid constantly increases, resulting in some abrasive particles being thrown out of the polishing region, which reduces the number of effective abrasive particles in the polishing region. Meanwhile, the increase in the working gap reduces the magnetic field strength in the polishing region, leading to a rapid decrease in the shear strength of the magnetic chains due to the reduced magnetic field strength. Figure 8b depicts the response surface of the interaction between the polishing tool speed and the abrasive particle size on the shear force when the working interval is 1 mm. It can be seen that under different abrasive particle sizes, as the polishing tool speed increases, the shear force exhibits an upward trend, and the larger the abrasive particle size, the smaller the amplitude of the increase in shear force with the polishing tool speed. Figure 8c represents the response surface of the interaction between the working gap and the abrasive particle size on the shear force when the polishing tool speed is 600 r·min^−1^. It can be observed that under different working gaps, the rate of change in shear force with the abrasive particle size varies. The smaller the working gap, the greater the rate of change in shear force with the abrasive particle size. This suggests that the smaller the working gap, the greater the influence of the abrasive particle size on the shear force. This is because as the working gap decreases, the magnetic field strength increases, resulting in an increase in magnetic force, ultimately leading to a greater influence of the abrasive particle size on the shear force.

#### 3.4.2. Influence on Surface Roughness

**Figure 9:**
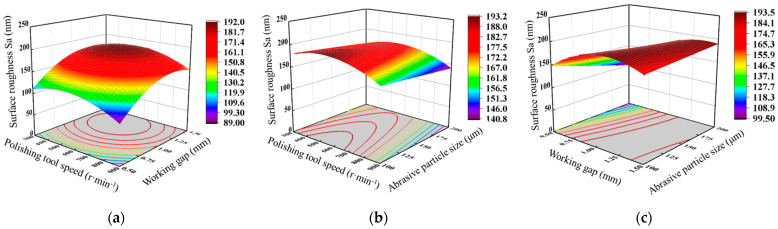
Response surface of the effect of processing parameters on surface roughness: (**a**) polishing tool speed and working gap; (**b**) polishing tool speed and abrasive particle size; (**c**) working gap and abrasive particle size.

Figure 9 depicts the interaction of the polishing tool speed, working gap, and abrasive particle size on surface roughness. Figure 9a shows the response surface of the interaction between the polishing tool speed and the working gap on surface roughness when the abrasive particle size is 150 μm. It can be observed that as the polishing tool speed increases, the surface roughness decreases gradually. As the polishing tool speed gradually increases, the material removal efficiency is improved, according to the Preston equation, which claimed that the material removal rate was positive for the polishing velocity. When the working gap is large, as the working gap decreases, the surface roughness decreases, and the magnitude of the decrease becomes larger. This is because as the working gap decreases, the magnetic field strength increases, resulting in an increase in magnetic force, which makes the cutting effect of abrasive particles on the workpiece more significant. Figure 9b illustrates the response surface of the interaction between the polishing tool speed and the abrasive particle size on surface roughness when the working gap is 1 mm. It can be seen that under constant abrasive particle size, as the polishing tool speed increases, the surface roughness first increases and then decreases. Figure 9c presents the response surface of the interaction between the working gap and the abrasive particle size on surface roughness when the polishing tool speed is 600 r·min^−1^. It can be observed that under a small working gap, as the abrasive particle size increases, the surface roughness decreases, whereas under a larger working gap, the surface roughness slightly increases with the increase in abrasive particle size. This is because the magnetic field strength is higher under a small working gap, which implies that the magnetic chains formed by magnetic particles can generate greater shear force on the workpiece surface, thereby enhancing the ability to remove surface roughness peaks. However, under a larger working gap, the magnetic field strength is lower, and the shear force generated by the magnetic chains formed by magnetic particles on the workpiece surface decreases rapidly. When using larger abrasive particles, the number of magnetic chains formed by the same mass of abrasive particles will decrease; fewer magnetic chains will form compared with using small particle sizes, resulting in a reduction in the removal chance of surface peaks [31]. Thus, although larger particles induce a size higher magnetic force acting on the surface, their ability to reduce surface roughness is not obvious compared with using small abrasive particles.

### 3.5. Verification

**Figure 10:**
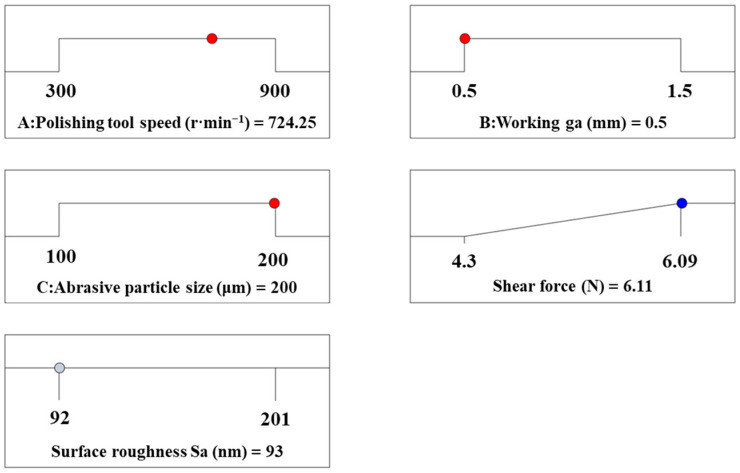
Maximum predicted *F*-value of the response surface model.

**Figure 11:**
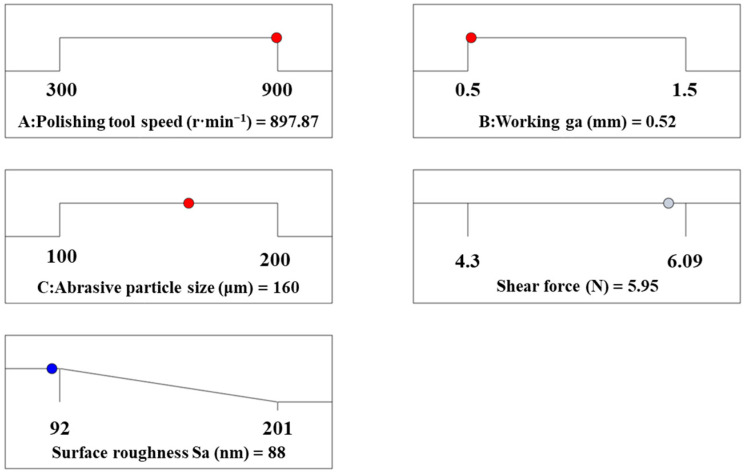
Optimal predicted surface roughness *S*_a_ of the response surface model.

The analysis of the response surface prediction model was conducted in this work, and the numerical function under the Optimization module [32] of Design Expert-13 was used for optimal calculation for both shear force and surface roughness. The optimal parameters for obtaining maximum shear force for rapid material removal were determined as follows: polishing tool speed of 724.254 r/min, working gap of 0.5 mm, and abrasive particle size of 200 μm. Under these conditions, the shear force was predicted to be 6.11 N, and the surface roughness *S*_a_ was 93 nm, as shown in Figure 10. Verification tests were conducted, and the average shear force of the three tests was 6.16 N, while the average surface roughness was 91 nm. Furthermore, the optimal processing parameters for minimum surface roughness for achieving the best surface quality were identified as: polishing tool speed of 897.87 r/min, working gap of 0.52 mm, and abrasive particle size of 160 μm. Under these conditions, the shear force was predicted to be 5.95 N, and the surface roughness was 88 nm, as shown in Figure 11. Verification tests were also performed based on these parameters, resulting in an average shear force of 6.03 N and an average surface roughness of 86 nm from the three tests. The close correlation between the verification test results and predicted values indicates the reasonableness of the response surface prediction model.

## 4. Conclusions

The magnetic field strength distribution of the Halbach array was simulated and verified in this paper. The polishing performance of the polishing tool was studied, and the surface morphology during polishing was also analyzed. With the response surface model, the optimal processing parameters were obtained. The conclusions are as follows:

(1) The magnetic field generated by the Halbach array exhibits periodic changes in magnetic field strength on the plane of the working gap. The distribution of the magnetic field strength was different as the radial diameter *R* increased.
(2) The polishing time has a stable material removal capability for the magnetic polishing tool. Additionally, the surface roughness improved deeply as polishing time increased. However, this polishing method can induce comet-tail polishing marks on the workpiece surface, which is a common defect in magnetic slurry polishing.
(3) The shear force and surface roughness can be affected to some extent by the combined action of the two process parameters. The correlation between the experimental results and predicted values indicates the reasonableness of the response surface prediction model.

## Figures and Tables

**Table 1:** Factors and levels of the response surface method.

Level	Factors		
	Revolution Speed of Carrier *n_c_* r·min^−1^ *A*	Working Gap *h* mm *B*	Abrasive Diameter μm *C*
−1	300	0.5	100
0	600	1	150
1	900	1.5	200

**Table 2:** Composition of magnetic abrasive particle slurry.

Carbonyl Iron Powder (wt.%)	Base Liquid (wt.%)	Cellulose (wt.%)
85	10	5

**Table 3:** Response surface experimental design plan and results.

Number	Factors			Result	
	*A* r·min^−1^	*B* mm	*C* mm	*x* N	*y* nm
1	300	0.5	150	5.31	112
2	900	0.5	150	5.72	92
3	300	1.5	150	4.70	164
4	900	1.5	150	5.13	142
5	300	1	100	4.30	183
6	900	1	100	4.83	168
7	300	1	200	4.92	161
8	900	1	200	5.43	132
9	600	0.5	100	5.12	139
10	600	1.5	100	4.57	173
11	600	0.5	200	6.09	104
12	600	1.5	200	4.47	201
13	600	1	150	4.53	192
14	600	1	150	4.54	187
15	600	1	150	4.47	183
16	600	1	150	4.67	178
17	600	1	150	4.83	173

**Table 4:** Variance analysis of the fitted equation for shear force.

Source	Sum of Deviation Square	Freedom Degree	Mean Square	*F*	*p*
Model	3.62	9	0.4022	12.75	0.0014
*A*	0.4418	1	0.4418	14.01	0.0072
*B*	1.42	1	1.42	45	0.0003
*C*	0.546	1	0.546	17.31	0.0042
*AB*	0.0001	1	0.0001	0.0032	0.9567
*AC*	0.0001	1	0.0001	0.0032	0.9567
*BC*	0.2862	1	0.2862	9.07	0.0196
*A*^2^	0.1809	1	0.1809	5.73	0.0479
*B*^2^	0.6728	1	0.6728	21.33	0.0024
*C*^2^	0.0126	1	0.0126	0.4001	0.5471
Residual	0.2208	7	0.0315		
Lack of fit	0.1379	3	0.046	2.22	0.2284
Pure error	0.0829	4	0.0207		
Total	3.84	16			

**Table 5:** Variance analysis of the optimized fitted equation for shear force.

Source	Sum of Deviation Square	Freedom Degree	Mean Square	*F*	*p*
Model	3.61	6	0.6012	25.73	<0.0001
*A*	0.4418	1	0.4418	18.91	0.0014
*B*	1.42	1	1.42	60.76	<0.0001
*C*	0.546	1	0.546	23.37	0.0007
*BC*	0.2862	1	0.2862	12.25	0.0057
*A*^2^	0.1864	1	0.1864	7.98	0.018
*B*^2^	0.6845	1	0.6845	29.3	0.0003
Residual error	0.2336	10	0.0234		
Lack of fit	0.1507	6	0.0251	1.21	0.4455
Pure Error	0.0829	4	0.0207		
Total	3.84	16			

**Table 6:** Reliability analysis of the shear force prediction model.

Model	Correlation Coefficient	Adjusted Correlation Coefficient	Predictive Correlation Coefficient	Variable Coefficient	Signal-to-Noise Ratio
	*R*^2^	*R*^2^*_Adj_*	*R*^2^*_Pre_*	*C.V.*%	*SNR*
Initial	0.9425	0.8686	0.3917	3.61	12.0672
Post-optimization	0.9392	0.9027	0.7227	3.11	16.7081

**Table 7:** Analysis of variance of the fitted equation for surface roughness.

Source	Sum of Deviation Square	Freedom Degree	Mean Square	*F*	*p*
Model	16,119.81	9	1791.09	19.23	0.0004
*A*	924.5	1	924.5	9.93	0.0161
*B*	6786.12	1	6786.12	72.86	<0.0001
*C*	528.13	1	528.13	5.67	0.0488
*AB*	1	1	1	0.0107	0.9204
*AC*	49	1	49	0.5261	0.4918
*BC*	992.25	1	992.25	10.65	0.0138
*A*^2^	2460.76	1	2460.76	26.42	0.0013
*B*^2^	4026.76	1	4026.76	43.24	0.0003
*C*^2^	27.92	1	27.92	0.2998	0.601
Residual	651.95	7	93.14		
Lack of fit	430.75	3	143.58	2.6	0.1896
Pure error	221.2	4	55.3		
Total	16,771.76	16			

**Table 8:** Variance analysis of the optimized fitted equation for surface roughness.

Source	Sum of Deviation Square	Freedom Degree	Mean Square	*F*	*p*
Model	16,041.9	6	2673.65	36.63	<0.0001
*A*	924.5	1	924.5	12.67	0.0052
*B*	6786.13	1	6786.13	92.98	<0.0001
*C*	528.13	1	528.13	7.24	0.0227
*BC*	992.25	1	992.25	13.59	0.0042
*A*^2^	2440.01	1	2440.01	33.43	0.0002
*B*^2^	4002.63	1	4002.63	54.84	<0.0001
Residual	729.87	10	72.99		
Lack of fit	508.67	6	84.78	1.53	0.3537
Pure error	221.2	4	55.3		
Total	16,771.76	16			

**Table 9:** Reliability analysis of the surface roughness prediction model.

Model	Correlation Coefficient	Adjusted Correlation Coefficient	Predictive Correlation Coefficient	Variable Coefficient	Signal-to-Noise Ratio
	*R*^2^	*R*^2^*_Adj_*	*R*^2^*_Pre_*	*C.V.*%	*SNR*
Initial	0.9611	0.9111	0.5685	6.11	13.8988
Post-optimization	0.9565	0.9304	0.7957	5.41	18.3624

## Data Availability

The original contributions presented in the study are included in the article, further inquiries can be directed to the corresponding author/s.
